# Selection of high affinity aptamer-ligand for dexamethasone and its electrochemical biosensor

**DOI:** 10.1038/s41598-019-42671-3

**Published:** 2019-04-29

**Authors:** Somia Mehennaoui, Sujittra Poorahong, Gaston Contreras Jimenez, Mohamed Siaj

**Affiliations:** Department of Chemistry, Université du Québec à Montréal, Montréal, Québec, H3C 3P8 Canada

**Keywords:** Biochemistry, Health care

## Abstract

A high specificity aptamer-ligand biorecognition and binding system to monitor of dexamethasone (DXN) was developed. The detection principle was based on a label-free electrochemical aptasensor. The selection of the aptamer was successfully performed by the systematic evolution of ligands through exponential enrichment technique (SELEX). From a random library of 1.08 × 10^15^ single-stranded DNA, an aptamer designated as DEX04 showed a highest affinity with a dissociation constant of 18.35 nM. It also showed a good conformational change when binding with DXN. In addition, the aptamer DEX04 did not show any cross-reactivity with other commonly used hormones. An impedimetric aptasensor for DXN was then developed by immobilizing DEX04 on a gold electrode. The binding upon to DXN was monitored by following the change in the charge transfer resistance (Rct) of the [Fe(CN)_6_]^4−/3−^ redox couple. The aptasensor exhibited a linear range from 2.5 to 100 nM with a detection limit of 2.12 nM. When applied aptasensor to test in water samples, it showed good recovery percentages. The new DXN aptamer can be employed in other biosensing applications for food control and the diagnosis of some diseases in medicine as a cost-effective, sensitive and rapid detection method.

## Introduction

Dexamethasone (DXN) is a synthetic hormone belonging to the group of corticosteroids. It is mainly used as an anti-inflammatory, anti-allergic and immunosuppressive agent in many medical applications^1^. Often, it was used as a growth promoting agent to increase the body mass^2^. The residues of DXN in meat and other animal products *i.e*., milk can be harmful to humans and animals^3,4^. With high concentrations, DXN can affect the nervous, endocrine and digestive systems of animals^5^. In addition, previous studies have shown that DXN can negatively influence fertility and ovarian function like chronic an ovulation and polycystic ovarian syndrome^6–8^. Based on animal studied It is classified as 1B category (presumed human reproductive toxicant). The upper dose should not more than 1,000 mg/kg by oral take^9^. Therefore, accurate monitoring of the existence and the concentration of DXN in environmental and clinical samples is very important. The concentrations of DXN in real samples were found from sub nanomolar to micromolar range with different matrices^1,10,11^. Thus, the development of a rapid and sensitive detection method for the identification of DXN is required.

Although, the immunological techniques, instrumental analytical approaches are generally costly, time-consuming and require highly qualified personnel, making them unsuitable for field applications^12,13^. To overcome these limitations, several studies have been devoted to the development of new and simple detection techniques. The biosensors have recently sparked considerable interest in a variety of biomedical and environmental applications and have emerged as an interesting alternative to conventional analytical and immunological assays. Recently, a photoelectrochemical immunosensor based on competitive strategy has been proposed for the detection of DXN^14^. Despite, their ease to use and sensitive detection, immunosensors are subject to a major challenge, precisely the use of antibodies as bioreceptor. Competitive immunoassays need additional steps and rely on enzymes for antibodies labeling. Furthermore, antibodies that target similar molecules are usually subject to cross-reactivity and thus require special storage and handling conditions. For that, aptasensors appeared as a good alternative to immunosensors because of their simple detection strategies^15^. Since their discovery in 1990^16,17^, aptamers offered many advantages, including *in-vitro* selection even for small molecules^18^, viruses^19^ and proteins^20^. They are also relatively easy to synthesize and modulate at low cost, highly stable at different storage conditions and transportation in ambient temperature.

Therefore, in this work we report a high affinity aptamer against DXN. The SELEX approach was employed for aptamer selection from a random library of 1.08 × 10^15^ single-stranded DNA. Herein we present the first report for selected aptamer for DXN detection with the highest affinity and good characterization results. In addition, the selected aptamer was integrated into a novel and simple label-free impedimetric platform based on gold electrodes. This novel aptasensor offers simple, low cost and highly stable detection method that can be used by unqualified personnel replacing currently sophisticated immunoassays and analytical methods.

## Results and Discussion

### *In vitro* selection of DNA aptamers for DXN

**Figure 1:**
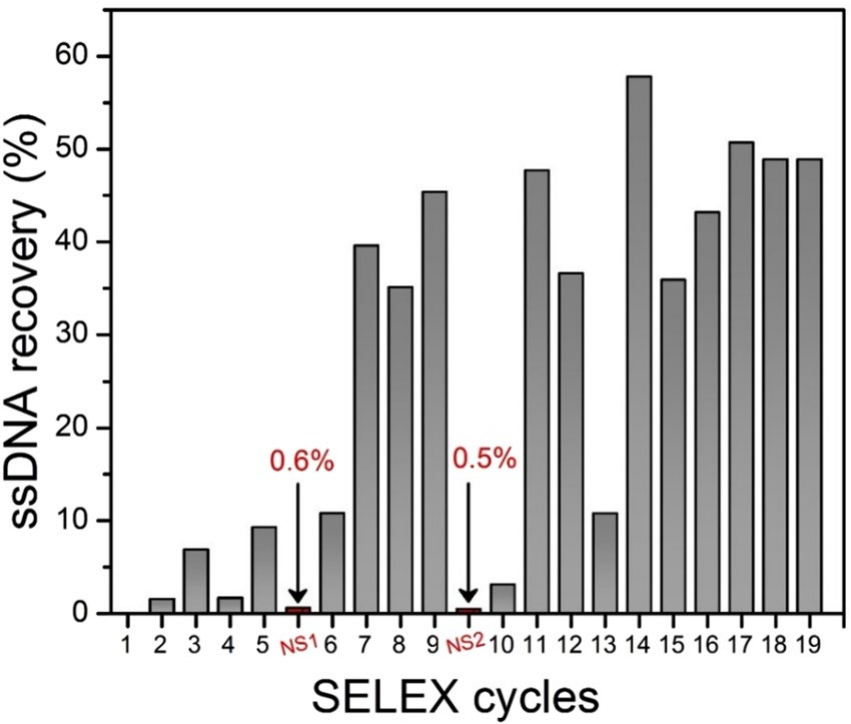
Selection of DXN aptamers; ssDNA recovery from each SELEX cycle and the resulting enrichment plateau. Negative selection (NS) performed by using blank beads.

Screening several aptamers that recognize DXN with a high affinity and specificity from a vast random library consisting of 1.08 × 10^15^ different ssDNA is a challenging task. To the best of our knowledge, no other aptamer-based biosensors were developed for the detection of DXN. In order to generate affinity complexes, the ssDNA pools were first incubated with DXN sepharose 6β beads. The process was carried out through 19 cycles of selection. Each SELEX round was controlled by measuring the percentage of eluted ssDNA (bound ssDNA) with respect to the initial concentration of the ssDNA quantified by fluorescence. As illustrated in Fig. 1, a low recovery percentage of ssDNA was observed in the first five cycles. This may be due to the low concentration of the used analytes or due to a low specifically bound aptamer to the DXN. Therefore, a negative selection (NS) was carried out using blank sepharose 6β beads in order to eliminate the non-specifically bound aptamers from the beads matrix. Hence, a significant increase in the recovery was detected in the next selection cycles. These also confirmed the important role of the counter or negative selection. In order to improve the selection accuracy and only keep the most specific DNA for free DXN, a second negative selection was performed before the tenth cycle. It was observed that a high amount of ssDNA was retained onto the blank beads, and a low amount of ssDNA, which expected to be a high specific binding to the analyte was obtained. In addition, a considerable rise in the ssDNA recovery was found in the next cycle, which indicates the specificity of this washed ssDNA for DXN. In order to retain the best affinity aptamers, therefore, after the 12^th^ cycle, the concentration of analyte was reduced. This makes the 13^th^ cycle shows low recovery. At the 19^th^ cycle, after reaching a plateau with a significant enrichment of DXN binding DNA, the ssDNA pool was cloned and sequenced.

### Characterization of selected aptamers

Ten positive white colonies were obtained after cloning processes. Among them only six colonies were obtained well-defined sequence. A significant consensus region of nucleotides was observed between the sequences belonging to the same group or family. These can be aligned into three families (A, B and C) as shown in Table 1 and all sequences were then characterized.

**Table 1:** Selected aptamer sequences against DXN.

Family	Name	Aptamer structure 5′ to 3′	*K*_*D*_ (nM)
A	DEX01	ACACCCCACGTAGTGTCACAGCACGCTTATAGTAAGTGAAGTGACGGGTTGCTGATGTG	15.71
	DEX03	ACGCGTAGGGATGTGTAAGGTCTGTACACCTCGGTTTACTCTATGCTTCGCATATTGTCG	72.83
B	DEX04	ACACGACGAGGGACGAGGAGTACTTGCCAACGATAACGTCGTTGGATCTGTCTGTGCCC	18.35
	DEX10	GGACAGCTGGCCGCGAAGCGAGACACGTATAAGGTACTATACGGCTGGCATATGTATCTG	715
C	DEX05	ACAGGCTTGGATTAGTGTATCCAACTAGTATCGTGTATACTAGGCCCTTGCTACCCTGTG	NB
	DEX06	ACACACGAAACACAAGCAGTGAGACTGCCTACGTCCGTAGTTGTGTTGAGTTTGCTCTCC	NB

**Figure 2:**
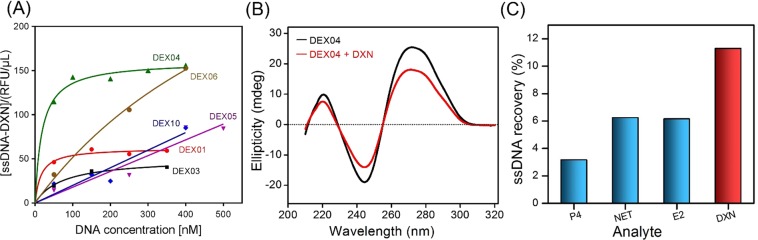
(**A**) Binding saturation curve of all aptamers with DXN beads determined by the fluorescence assay, by plotting the concentration of the complex formed by the binding between ssDNA aptamer and DXN analyte ([ssDNA−DXN]) as a function of unbound ssDNA concentration. (**B**) Circular dichroism spectra of 3 μM of the DEX04 before (Black line) and after recognition of 3 μM DXN (Red line). (**C**) Cross-reactivity study of the DEX04 aptamer to progesterone (P4), norethisterone (NET) and 17β-estradiol (E2).

After sequencing, the affinity binding by fluorescence assay was determined. The binding curves of all aptamers are shown in Fig. 2A. The dissociation constant values were then calculated using nonlinear regression. Only four aptamers provided a good affinity to DXN in a low nanomolar level with K_D_ ranging from 15 to 715 nM (Table 1). This is suggesting a strong affinity binding between aptamer and DXN occurred. Whereas the two aptamers, *i.e*., DEX05 and DEX06 did not exhibit binding against DXN. The four aptamers which provided the affinity binding to DXN were then studied the conformation changes in the structure before and after binding to DXN by circular dichroism (CD) spectroscopy. From our results, DEX04 shows the most significant change in ellipticity as shown in Fig. 2B. In contrast, the other three aptamers; DEX01, DEX03 and DEX10, did not show a significant ellipticity change as shown in Fig. S1. In detail, the CD spectrum of the free DEX04 aptamer has a positive peak at 271 nm and a negative peak at 244.5 nm (Black line), which is a duplex type characteristic. Following the binding and biorecognition of the DEX04 aptamer to DXN, a significant change of ellipticity at 271 nm and 245.5 nm was observed (red line). This indicates the folding of the aptamer into other conformations following its contact with the molecule of DXN, which contains chiral atoms and could divert polarized light circularly.

Then the specificity of DNX aptamer, cross-reactivity assays with progesterone (P4), norethisterone (NET) and estradiol (E2) hormones which are DXN’s analogs and can present in the same environmental samples was studied. As shown in Figs 2C and S3, the DEX04 indicates the highest selectivity to DXN against P4, NET and E2 hormones. Anti-DXN aptamers have been further investigated by studying their secondary structures. As depicted in Fig. S3, all aptamer sequences exhibited formation of characteristics of stem-loop shape. Those structures showed good binding affinity with DXN as confirmed the presence of correspondences and differences. Structural similarities are observed which is consistent with aptamer recognition of the target conformation. From all characterization results, DXN 04 provided the best performance, in terms of good *K*_D_, specificity and change in conformation. Hence, it was chosen for further biosensor applications.

### Electrochemical Aptasensor of DXN Detection

**Figure 3:**
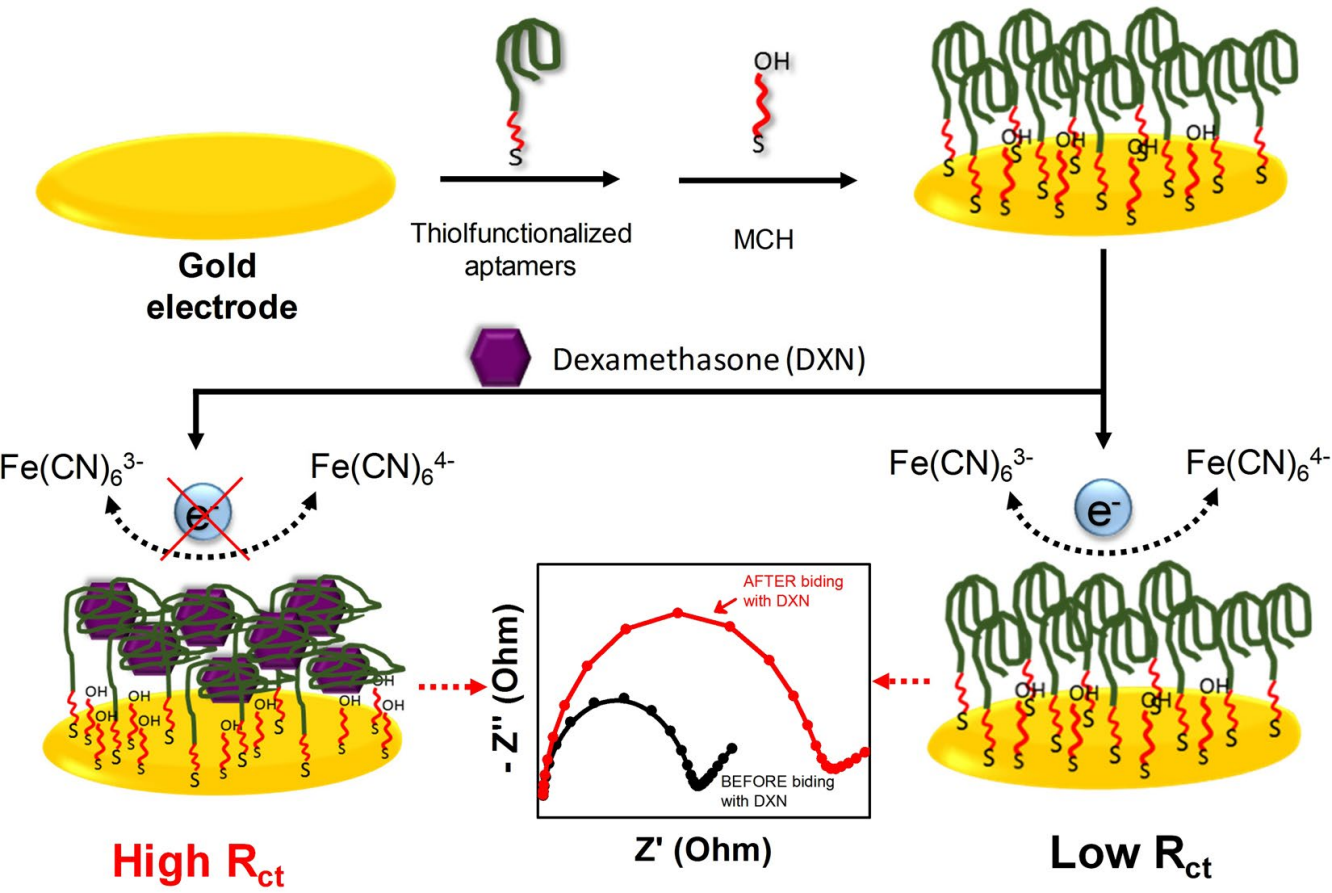
Impedimetric mechanism of aptasensor.

**Figure 4:**
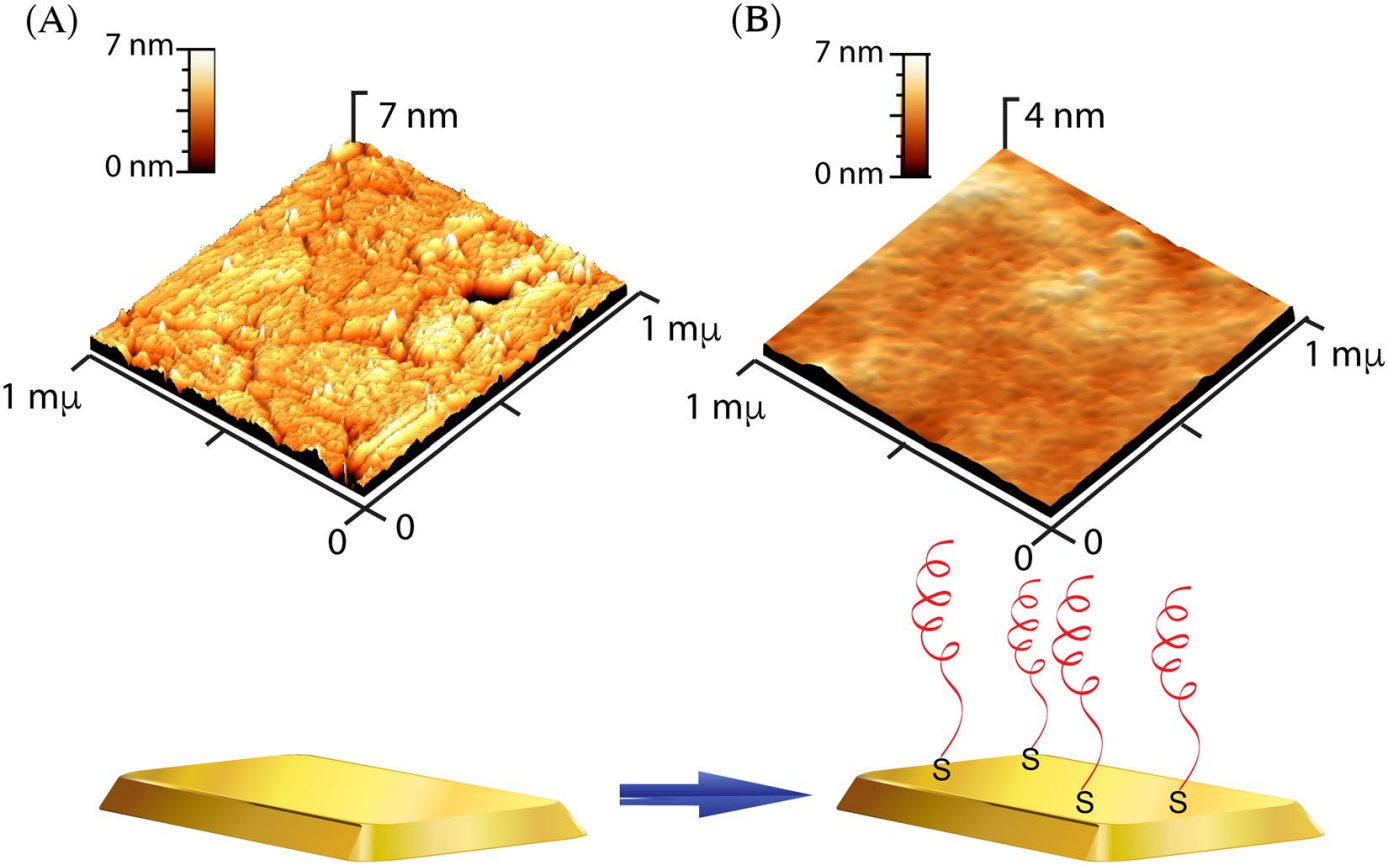
(**A**) AFM images of the bare electrode and (**B**) DEX04 aptamer-immobilized gold electrode.

**Figure 5:**
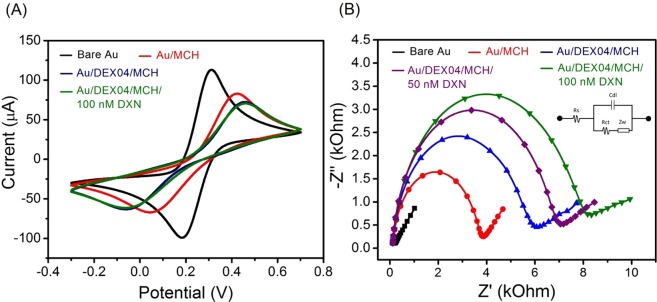
(**A**) Cyclic voltammograms and (**B**) impedance spectra (Nyquist plots) of 10 mM [Fe(CN)_6_]^4−/3−^ in PBS, pH 7.4, for bare Au electrode, Au/MCH, Au/MCH/DEX04 before and after recognition to 50 nM and 100 nM DXN. The inset is the equivalent circuit used for the impedance data fitting; R_s_ is the solution resistance between working and reference electrodes, Z_w_ is Warburg impedance; Cdl is the double layer capacitance and R_*ct*_ is the charge-transfer resistance.

For the DXN aptasensor, the DEX04 aptamer sequence (5′-ACA CGA CGA GGG ACG AGG AGT ACT TGC CAA CGA TAA CGT CGT TGG ATC TGT CTG TGC CC-3′), which revealed a significant conformation change in the CD measurement and provided a low *K*_D_ value of 18.35 nM by fluorescence, was employed. Figure 3 demonstrates the different steps to fabricate the DXN aptasensor. First, the disulfide-modified aptamer (HOC6-S-S-C6-DEX04) was immobilized on a gold surface by self-assembly and the free gold surfaces were blocked using MCH in order to minimize the nonspecific adsorption of the aptamers and ensure that the aptamer binds only from the sulfide side. Atomic force microscopy (AFM) was used to characterize the morphology of the gold electrodes surface before and after the aptamer immobilization (Fig. 4). A comparison between the AFM images of bare and modified electrode shows clearly the aptamer immobilization. The formation of a complete aptamer monolayer on top of the gold electrode induces a significant decrease in the roughness of the electrode. Before the aptamer immobilization, the bare gold electrode showed a roughness value of 7 nm compared to 4 nm the chemisorbed DEX on the electrode surface. In addition, the resulting modified electrodes have been characterized by X-ray photoelectron spectroscopy (XPS) and Attenuated total reflection-Fourier transform infrared (ATR-FTIR). Compared to bare gold electrode, the high-resolution XPS spectra of sulfur (S) for modified electrodes showed a S 2p peak at 162.4 eV. This peak is attributed to a Au-S bond indicating that the chemical grafting of the thiol-modified aptamer to the gold surface was successful^21^ (Fig. S4). However, from the ATR-FTIR data we can conclude that we have the ssDNA attached to the electrode surface^22,23^ (Fig. S5). All the steps of the fabrication of the aptasensor were controlled by EIS and CV measurements. As shown in Fig. 5A, the characteristic ferricyanide redox peaks resulting from the bare gold electrodes declined gradually after being exposed to MCH, free DEX04 aptamer and DEX04 bound to DXN. As anticipated, the bare gold electrode showed a quasi-reversible voltammogram of the ferricyanide redox couple with a peak separation ΔEp of 120 mV (black curve). After modification with MCH (red curve), the electrochemical reaction is blocked on Au electrode surface, which leads to an increase in the peak separation and a substantial decrease in the peak current. Once the Au electrode incubated with the aptamer and followed by MCH (blue curve), a higher degree of decreasing in the electron transfer charge between the redox couple and the electrode were observed compared to Au electrode modified with MCH only. This could be affected by the generation of a negatively charged DNA that rejects the [Fe(CN)_6_]^4−/3−^ anions and retards the interfacial kinetics of the redox couple at the Au interface. Thus, this is indicating the successful attachment of the aptamer on the gold surface. Moreover, the peak current reflects an additional decrease of the electron-transfer rate after incubation with 100 nM DXN (green curve), confirming the success of binding between DXN and the aptasensor.

Likewise, ESIs for each step of the electrode modifying in 10 mM [Fe(CN)_6_]^4−/3−^ solution as probe redox were recorded (Fig. 5B). Impedance measurements were represented by Nyquist plots. These plots consist of a part forming semicircles at high frequencies corresponding to electron transfer and a straight line at very low frequencies representing the diffusion processes. The quantification of the semicircle diameter can be fitted with the Randles-modified equivalent circuit or the electron transfer resistance (R_et_) of the modified electrode surfaces. The obtained ESIs data corroborate the preceding results obtained by CV. More specifically, the EIS corresponding to the bare gold electrode presents a small semicircle diameter (black curve) indicating the rapid electron transfer and diffusion limiting processes on the bare gold electrode surface. After incubating with MCH, a preventing of the electrochemical reaction occurred, an increase in the charge transfer resistance (R_ct_) was noticed. After immersing the gold electrode in the disulfide-modified aptamer and followed by MCH blocking, a significant increase in the diameter of the semicircle was observed indicating the increase in the R_ct_ caused by the assembly of a negatively charged disulfide DNA which repels the [Fe(CN)_6_]^4−/3−^ anions of the redox couple on the electrode surface, which confirms the success of the self-assembly. We notice a significant increase in R_ct_ (7810 Ω) after incubating the aptasensor with 50 nM of DXN. This R_ct_ change may be resulting from the conformation changes of the aptamer upon binding to DXN, which leads to more shielding of the Au surface and more retardation for the [Fe(CN)_6_]^4−/3−^ anions accessibility to the surface electrode. When DXN concentration increased to 100 nM, a further small increase in R_ct_ was observed. Both of CV and EIS results confirm that the sensing interface is achieved successfully.

**Figure 6:**
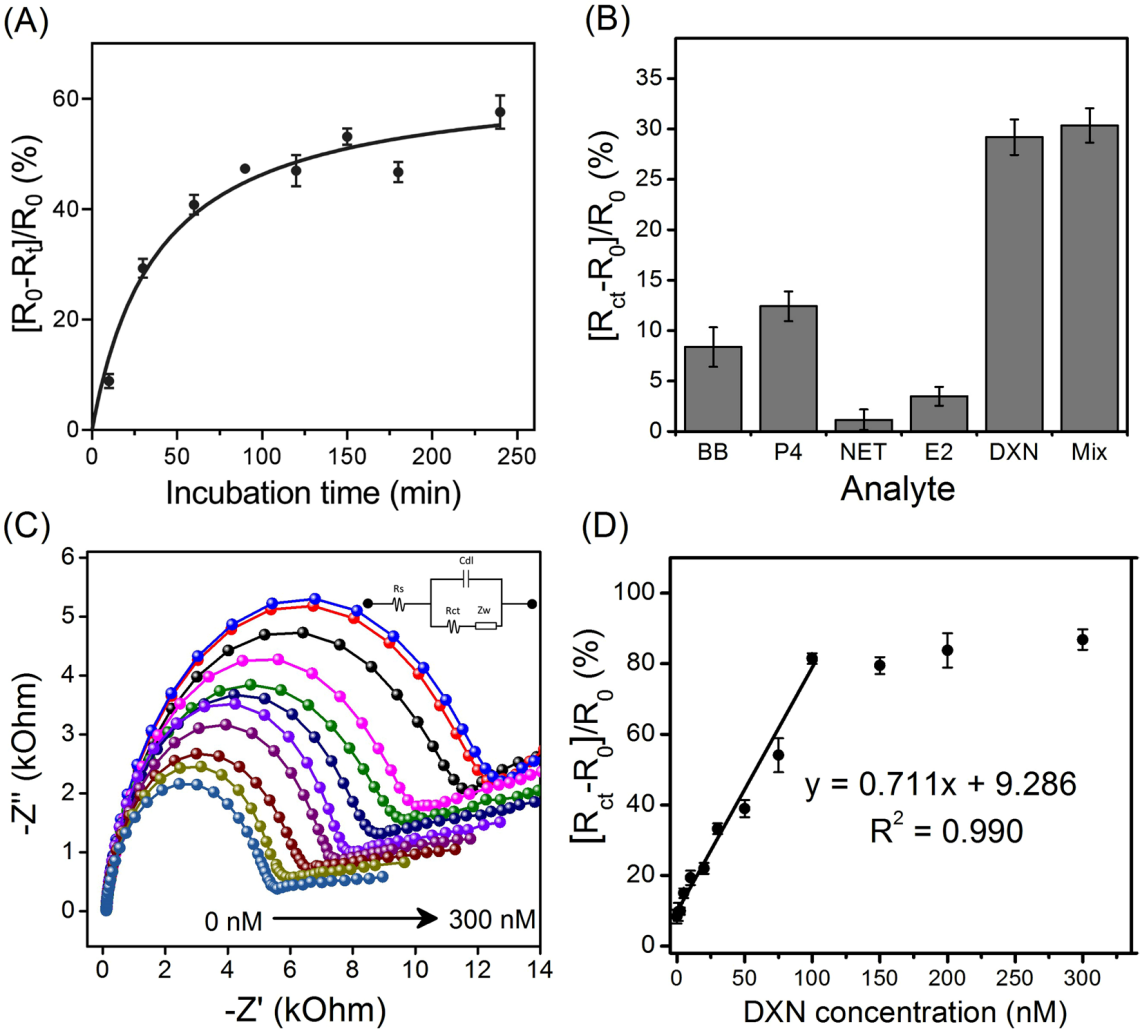
(**A**) Effect of the DXN incubation time on aptasensor’s response for 50 nM DXN, (**B**) The cross reactivity of the DXN aptasensor against binding buffer (BB), 1 nM of DXN, P4, NET, E2 and their mixture, (**C**) Examples of Nyquist plots measurements in 10 mM [Fe(CN)_6_]^4−/3−^ of the aptasensor in different concentrations of DXN analyte (0, 1, 10, 20, 30, 50, 75, 100, 150, 200 and 300 nM). (**D**) Calibration curve of the aptasensor plotted with a regression coefficient r^2^ = 0.99.

The incubation time is a crucial factor, which could affect the performance of the aptasensor in DXN recognition. The incubation time was studied by incubating the DEX04 aptamer immobilized on Au electrode with 50 nM DXN at various duration. To evaluate the aptasensor’s response, we used the percentage change in the R_ct_ before and after binding to DXN. As shown in Fig. 6A, the aptasensor’s response increased by increasing the incubation time from 10 to 100 min. A slight increase in the sensor’s response was also observed from 100 to 240 min, indicating the saturation of the aptamer modified electrode. Consequently, the aptasensor was incubated for 120 min in all future experiments. The incubation time of our sensor is longer than the immunosensor maybe because in immunosensor system the anti-dexamethasone is incubated on TiO_2_ nanoparticles^14^. These nanoparticles have a larger surface area than our gold base electrode. It supports the dexamethasone easily to excess to the anti-dexamethasone bead.

The selectivity of the DXN aptasensor was studied in the presence of other hormone analogs to DXN. To achieve this, 1 nM of DXN, P4, NET, E2, binding buffer and their mixture were tested. Figure 6B presents the bar chart of the sensor’s response of (R_0_ − R)/R_0_, which demonstrates the high response obtained with the DXN and the mixtures compared to the nonsignificant changes in the case of the other components introduced, thus indicating the selectivity and the specificity of the developed aptasensor to DXN. The calibration curves of DXN were then constructed by monitoring the sensor’s response (Fig. 6C). Then the calibration curves of DXN were then constructed (Fig. 6D). Compared with different detection methods (see Table 2), the developed aptasensor exhibited excellent analytical performance with a wide linear range of 2.5 to 100 nM and low detection limit at 2.12 nM, calculated by IUPAC method^24^. The error bars represent the standard deviation SD of the three measurements.

**Table 2:** Figures of merits of comparable methods for determination of dexamethasone.

Method	Linear dynamic range	LOD	Applications/Comment	Ref.
Label-free electrochemical aptasensor	2.5–100 nM	2.12 nM	Applied to tab water and drinking water, recovery = 81.5–103.2%	This work
reverse-phase high-performance liquid chromatography with diode array detection	Not report	water = 6 ng/mL Feed = 190 ng/g	Applied to water and feed for meat-producing animals: recovery = 99.4 ± 1.3% Flumethasone was used as internal standard	^3^
Electrochemical sensor based Fe_3_O_4_/PANI–Cu^II^ microsphere	0.05 to 30 mM	3.0 nM	Applied to human urine and serum samples Recovery = 97.0–102.0%	^11^
high-performance liquid chromatography with diode array detection	Not report	10 ng/mL	Applied to human plasma with recovery = 96.96% to 106.07%	^27^
Hanging mercury drop electrode	25.5–122.3 µM	7.6 µM	Applied to drug sample; recovery = 99.8–100%	^28^
square-wave adsorptive voltammetry	0.0498–0.61 µM	2.54 nM	Applied to eye drops, injectable, elixir; recovery = 94.14–112.41%	^29^

### Application of DXN aptasensor in real simples

To evaluate the performance of the developed aptasensor for the detection of DXN in real samples, series of experiments have been carried out using tap water, drinking fountain water and ultrapure water with three different concentrations of DXN varying from 0 nM, 50 nM and 100 nM. Table 3 presents the results obtained for each sample. The negative water samples in binding buffer did not exhibit any significant signal. Good percentages of recovery ranging from 81.5% to 103.2% were obtained indicating no effect of interference from water components on the aptasensor’s detection mechanism. The relative differences are between 2.2% and 11.5%. These shown an acceptable recovery rate in levels of ng/mL is between 40 and 120% and the RSD could be 30% to 45%^24^. Thus, it can be concluded that recoveries for all concentrations of DXN in the studied samples are in the acceptable range. Finally, these results approved the actual application and the possible use of the impedimetric aptasensor in environmental analysis.

**Table 3:** Spike and Recovery Results from the application of DXN Aptasensor in real samples.

Sample	Spiked DXN (nM)	[R_et_ − R_0_]/R_0_ (%)	Recovery (%)	RSD (%)
**Tap water**	0	7.1 ± 2.3	—	2.26
	50	46.0 ± 2.5	103.29	6.93
	100	73.6 ± 1.6	90.68	2.19
**Drinking water**	0	14.3 ± 4.7	—	4.67
	50	38.3 ± 2.3	81.55	6.45
	100	77.7 ± 8.2	96.19	11.57
**Ultrapure water**	0	10.6 ± 2.4	—	2.39
	50	30.5 ± 3.3	88.03	8.13
	100	76.5 ± 3.6	94.54	5.05

## Conclusion

In this work, we have selected, identified and characterized the first aptamer with a high affinity and specificity to dexamethasone, a widely used hormone with low molecular weight (392.46 Da). The developed aptamer DEX04 presented a dissociation constant in the range of nanomolar range and demonstrated a significant change in its conformation before and after DXN recognition as shown by circular dichroism spectroscopy. The DEX04 aptamer was then integrated to design a label-free impedance based aptasensor. The aptasensor had a low LOD of 2.12 nM with a wide linear range of 2.5 nM to 100 nM and a good yield in the range of 81.5 to 103.2% as demonstrated by the application performed on different water samples. Other applications for the selected DXN aptasensor in meat, milk and some clinical samples will be studied in the future.

## Experimental Section

### Materials and reagents

The materials and reagents used for the selection, characterization of the aptamer and the development of the DXN aptasensor are cited in the supplementary information section.

### *In Vitro* Selection of DXN aptamers

The selection of aptamers was done through the SELEX process in which DXN conjugated sepharose 6β beads were exposed to the ssDNA library. This study, a random library containing 1.08 × 10^15^ oligonucleotides was designed. It is composed of a principal randomized part of 60 nucleotides connected by two constant primer-hybridization sites at the 3′ and 5′ extremities (5′-ATATCATATGCTCCAATT-N60-ATATTACACTTGCGATCT-3′). The primers were labeled with fluorescein in order to determine the recovered ssDNA and hexaethylene glycol (HEGL) linker to inhibit the polymerase prolongation. Five successive steps were accomplished for each aptamer selection and amplification cycle as shown in Fig. S6. Specifically, a 100 pmol ssDNA pool (1.794 nmol at the first cycle), measured by UV spectrophotometer was pretreated by heating at 90 °C for 5 min followed by renatured at 4 °C for 10 min. Then the mixture was left at ambient temperature for 5 min before adding 50 μL for the first cycle (100 μL for the second round and beyond) of prewashed DXN sepharose 6β beads in 400 μL binding buffer in a centrifuge filter tube. The solution was then incubated for 2 hours at room temperature. After incubation, the beads were washed several times with the binding buffer until no ssDNA is detected by fluorospectrometer (Nano Drop 3300, Fisher Scientific, Canada). The ssDNA aptamers bound to DXN sepharose beads were then eluted 6 times with 250 µL of elution buffer and heated at 90 °C for 10 minutes. Each eluting time, the elution was measured by fluorospectrometer until no DNA is detected. Eluted ssDNA was then concentrated and desalted by ultrafiltration using a 3 kDa cut off membrane.

The eluted DNA was amplified by Polymerase Chain Reaction (PCR). PCR products were concentrated by Speed Vac and re-suspended in 50:50 v/v of water:formamide and later heated at 55 °C for 5 min. The fluorescein labeled DNA was then isolated from the double-stranded PCR product by loading into 12% denaturing polyacrylamide gel electrophoresis (PAGE). Then it was eluted from the gel band through freeze thaw cycle. Eluted ssDNA from in the TE buffer (10 mM Tris pH 7.4, 1 mM EDTA) was concentrated by the ultrafiltration and then quantified by UV to be used for the next selection round. During SELEX cycles, two negative selection rounds were performed before the 6^th^ and 10^th^ cycles. This is carried out by incubating the DNA pool with blank sepharose beads. In this counter selection step, washed ssDNA were collected and exposed to the same pre-treatment mentioned previously.

### Cloning and sequencing of selected DNA

The selected ssDNA from the latest SELEX round were amplified by PCR using non-labeled primers. Afterwards, the DNA was cloned into pCR2.1-TOPO vector with the TOPO TA Cloning Kit (Invitrogen) using the competent cells *E. coli*. The bacteria that contained the plasmids were then cultured on petri dishes containing LB-agar medium enriched with 200 μL of ampicillin (20 mg/mL) and 100 μL of IPTGX-GAL. Positive white clones which contain a single sequence of ssDNA inserted into their plasmid were picked from petri dishes for subsequent cultivation in tubes containing liquid media. After incubation and growth, the ssDNA was amplified using the M13 forward and reverse primer sites within the vector and purified by QIAquick PCR. Finally, the ssDNA selected by the SELEX process were analyzed and aligned using the DIALIGN software^25^. Analysis of the secondary structure of identified aptamers was performed using the internet-free Mfold software^26^.

### Determination of dissociation constant and cross-reactivity Study by fluorescence assay

A high affinity binding between aptamers and DXN is a critical requirement for biosensing purposes. A binding assay was determined by studying the dissociation constant (K_D_). The identified aptamer sequences were amplified by PCR and labeled with fluorescein fluorophore. They were pretreated and then incubated in a 20 µL of DXN beads and various concentrations of the aptamers, *i.e*., 50, 100, 200, 300 and 400 nM. After heating and cooling treatment, the mixtures were washed and DNA was eluted. From each eluted DNA sequences, the fluorescence signal was quantified. A saturation curve was plotted and the *K*_*D*_ values were respectively calculated using the nonlinear regression method.

Cross-reactivity refers to the ability of a given aptamer to react with other analytes or hormones which have a similar chemical structure to DXN, *i.e*., progesterone (P4), norethisterone (NET) and estradiol (E2). After amplifying aptamers by PCR, a 150 nM of each aptamer was incubated with 20 µL P4 beads (a load capacity of 4.4 nmoL/mL) and 5 µL of the NET, E2 and DXN beads (loading capacity ligands of 6–14 μmoL/mL). The percentage binding of each hormone with the aptamers was determined by measuring the elution DNA using NanoDrop 3300 fluorospectrometer.

### Circular Dichroism (CD) study

The conformational changes in the structure of the aptamer upon DXN recognition were studied by circular dichroism spectroscopy. The spectrum of each sequence was analyzed before and after adding 3 μM of DXN into 3 μM of aptamer sequences. To obtain accurate results, we recorded and subtracted the background signals of binding buffer and 3 μM DXN from the CD spectra. The CD measurements were performed using Jasco-810 spectropolarimeter. Each CD spectrum was collected from 200 to 320 nm wavelengths at 0.1 nm intervals and an accumulation of three scans at 20 nm/min, with a 1 nm bandwidth and a time constant of 1 second.

### Electrochemical detection of DXN

In this work, gold (Au) rod electrode was used as a basis surface for modified of the aptamers. The immobilization is based on self-assembly of a disulfide-aptamer on Au surface. Before using Au electrodes (2.0 mm diameter) were cleaned and polished with 1, 0.3 and 0.05 µm alumina slurries (Al_2_O_3_). The electrodes were then washed and sonicated for 2 min in ultrapure water and subsequently immersed for 1 min in a piranha solution (3:1 mixture of concentrated H_2_SO_4_ and 30% H_2_O_2_), then washed again with ultrapure water and sonicated for 2 min in 100% ethanol. The electrodes were then electrochemically treated by cyclic voltammetry in 0.5 M of H_2_SO_4_ by cycling a potential of 0 to +1.6 V with the scan rate of 100 mV/s for 15 scans. After washing with ultrapure water and dry with nitrogen, the electrodes were incubated in 1 μM disulfide-modified DEX04 aptamer (the best performance aptamer) in the binding buffer for 24 hours at room temperature. After that, the DEX04 aptamer-modified electrodes were cleaned with the binding buffer to remove any unbound aptamers. The electrodes were then immersed in a 1 µM 6-mercapto-1-hexanol (MCH) in 10 mM phosphate buffer saline, pH 7.4 for 30 min in order to block the remaining bare surfaces and to reduce the density of the aptamer layers by the displacement of the aptamers non-specifically adsorbed. At the end, the modified electrodes were thoroughly washed with the binding buffer and 1 M NaCl solution. For further uses, the modified electrodes were stored in the binding buffer at 4 °C.

For preliminary conditions of the detection, the aptamer-modified gold electrodes were incubated with 50 nM and 100 nM of DXN for 100 min. After washing with binding buffer to eliminate the unbound aptamers to DXN, the electrodes were subjected directly to record cyclic voltamograms and impedance spectra. For the electrochemical measurements, they were carried out by using SP-300 potentiostat (Bio-Logic Science Instrument, France) connected to a personal computer and driven by EC-Lab program. A frequency ranges from 100 kHz to 50 mHz using an alternative voltage with an amplitude of 10 mV, superimposed on a DC potential of 0.21 V (*vs* a Ag/AgCl reference electrode) was programed. All measurements were carried out in 10 mM PBS buffer, pH 7.4, in the presence of 10 mM [Fe(CN)_6_]^4−/3−^ as a redox couple.

## Supplementary information

Supp Info
