# Supplementary material for: Selection of high affinity aptamer-ligand for dexamethasone and its electrochemical biosensor
Source: Sci Rep. 2019 Apr 29;9:6600. doi: 10.1038/s41598-019-42671-3 (PMC6488579; doi:10.1038/s41598-019-42671-3)
Supplement: Supplementary file 1 — Supp Info [file 41598_2019_42671_MOESM1_ESM.pdf]

Supplementary information

Selection of high affinity aptamer-ligand for dexamethasone and its electrochemical biosensor

Somia Mehennaoui[1], Sujittra Poorahong[1], Gaston Contreras Jimenez[1], Mohamed Siaj[1*]

[1]Département de Chimie et Biochimie, Université du Québec à Montréal, Montréal, Québec H3C 3P8, Canada.

*Corresponding author: Email: siaj.mohamed@uqam.ca

Results and Discussion

Characterization of selected aptamers

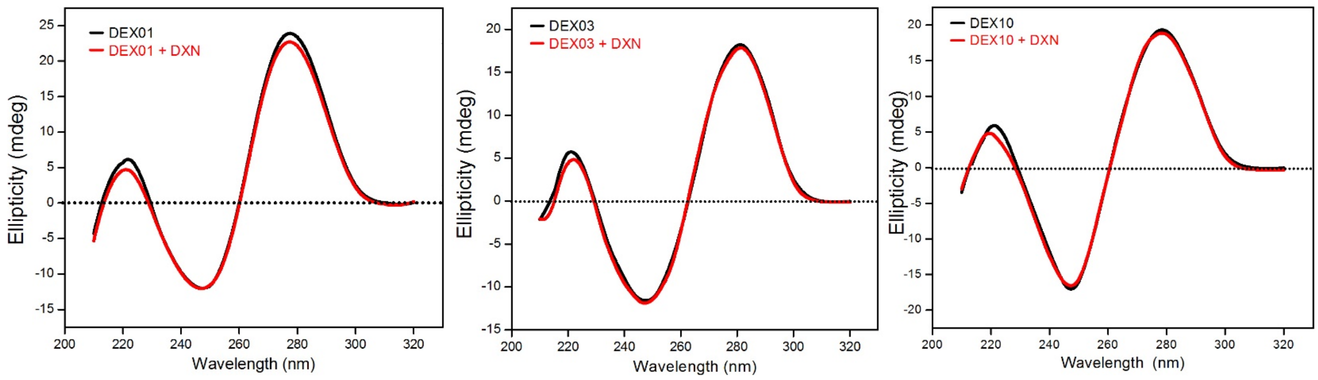

Supplementary Data Figure S1 Circular Dichroism spectra of 3 µM of DEX01, DEX03 and DEX10 aptamers before (Black line) and after binding (Red line) to 3 µM of DXN analyte.

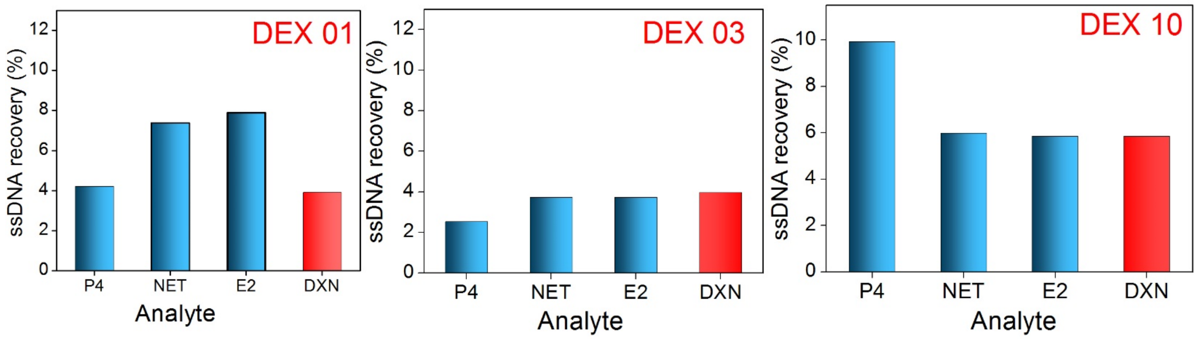

Supplementary Data Figure S2 Study of the cross-reactivity of the sequences DEX01, DEX03 and DEX10 by fluorescence assay.

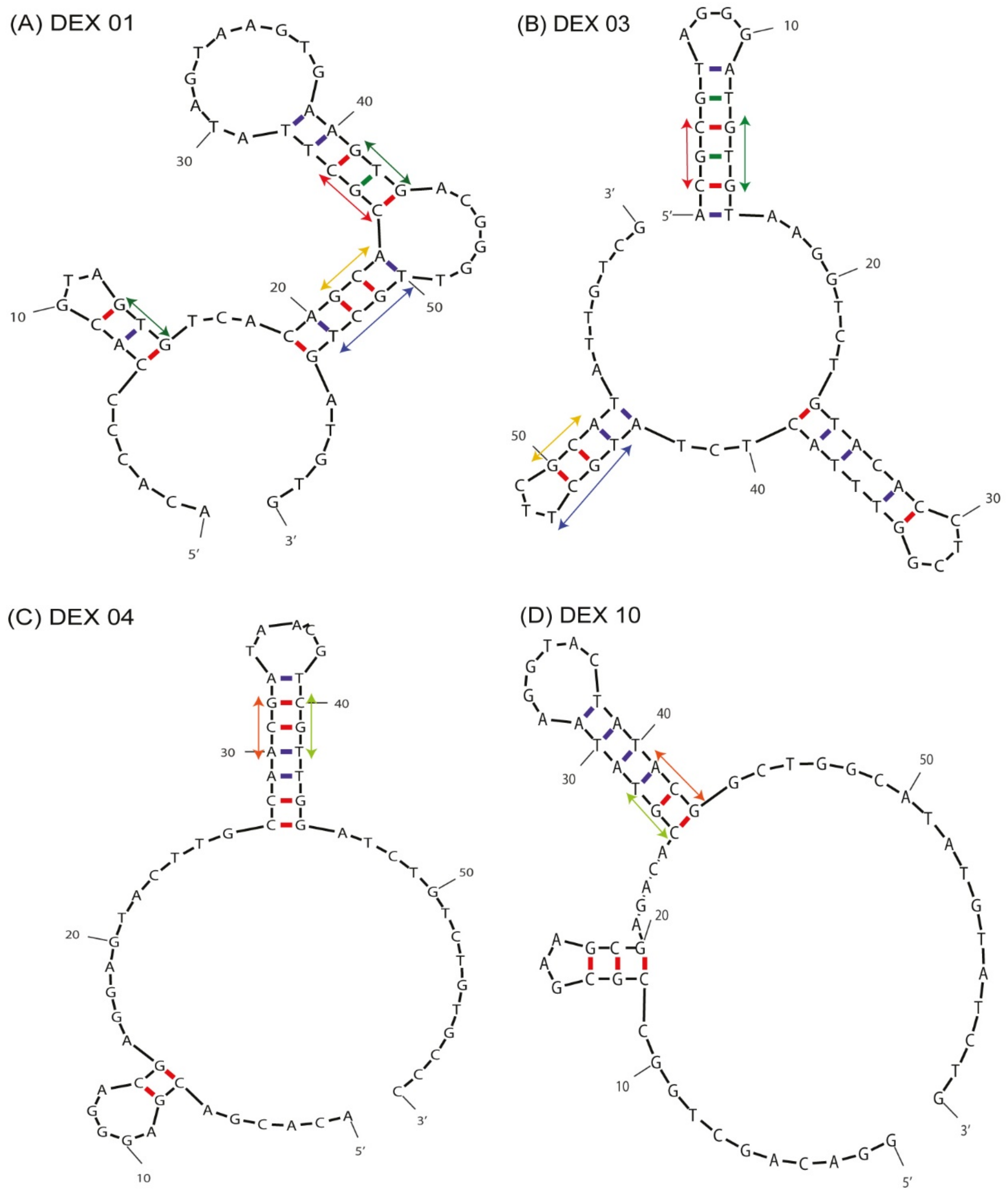

Supplementary Data Figure S3 Predication of secondary structures for DXN 01, 03, 04, 10 aptamers binding using the Mfold program based on a free energy minimization algorithm.

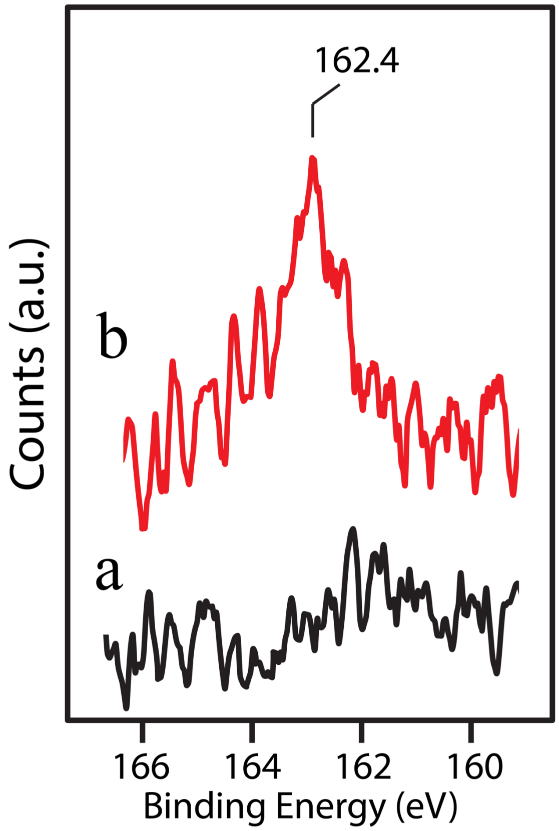

Supplementary Data Figure S4 (a) XPS high-resolution spectra of sulfur (S) and (b) bare Au electrode. The S 2p peak at 162.4 eV is attributed to a Au–S bond that show that the linking between the thiol-modified aptamer and the gold surface occurs (a), which is absent for the bare electrode (b).

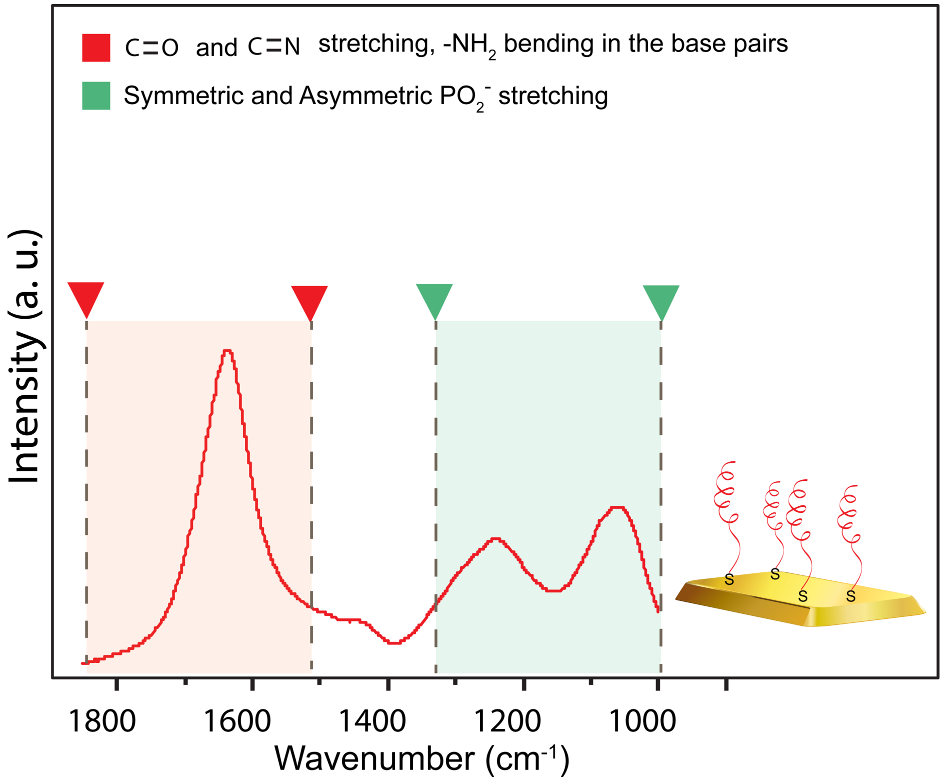

Supplementary Data Figure S5 ATR-FTIR spectra of self-assembled monolayers of the developed aptamer (DEX04) on the gold electrode surface. The ssDNA spectrum shows the characteristic peaks of the ssDNA bases.

Experimental Section

Materials and reagents. The ssDNA library, set of primers for polymerase chain reaction (PCR), unlabeled selected aptamer and the disulfide terminated aptamer sequence (HPLC purified) were custom-synthesized by Integrated DNA Technologies Inc. (Coralville, USA). Taq plus DNA polymerase and dNTP's for PCR, acrylamide/bisacrylamide (40% solution), urea, Tris-base, ammonium persulfate (APS) and Tetramethylethylenediamine (TEMED), boric acid and ethanol were purchased from Bioshop Inc. (Ontario, Canada). Dexamethasone (DXN), progesterone 3-O-carboxymethyloxime (P4 3-O-CMO), 17β-estradiol (E2), norethisterone (NET), phosphate-buffered saline pH 7.4 (PBS), potassium ferrocyanide ($K_4Fe(CN)_6$), potassium ferricyanide ($K_3Fe(CN)_6$), magnesium chloride ($MgCl_2$), sodium nitrite ($NaNO_2$), hydrochloric acid (HCl), sulfuric acid (H_2SO_4), and hydrogen peroxide (H_2O_2) were purchased from Sigma (Oakville, ON,

CAN). Dexamethasone-coupled 20 sepharose 6β beads, norethisterone-coupled 20 sepharose 6β beads and estradiol-coupled 20 sepharose 6β beads were purchased from Molecular 21 Targeting Technologies Inc. (West Chester, PA, USA). TOPO TA cloning kit with One Shot MAX Efficiency DH5α- 3 1 T1*E.coli* was purchased from Invitrogen (Burlington, ON, CAN). DNA purification kit QIAquick PCR was purchased from Qiagen (Toronto, ON, CAN). Amicon Ultra-0.5 mL centrifugal desalting filters with a 3 kDa molecular cut-off were obtained from Fischer Scientific (Ottawa, ON, CAN). Centrifuge tube filters with a cellulose acetate membrane with pore size of 0.45 µm were purchased from Corning life sciences (Tewksbury, 6 MA, USA). Binding buffer was used during the aptamer selection as well as in the electrochemical experiments consists of 50 mM Tris, 150 mM NaCl, 2 mM $MgCl_2$, pH 7.5. Eluting buffer is 7 M urea in binding buffer. Tris−EDTA buffer (TE buffer) is 10 mM Tris and 1 mM EDTA, pH 7.4. All solutions were prepared by Milli-Q grade water.

In Vitro Selection of DXN aptamers.

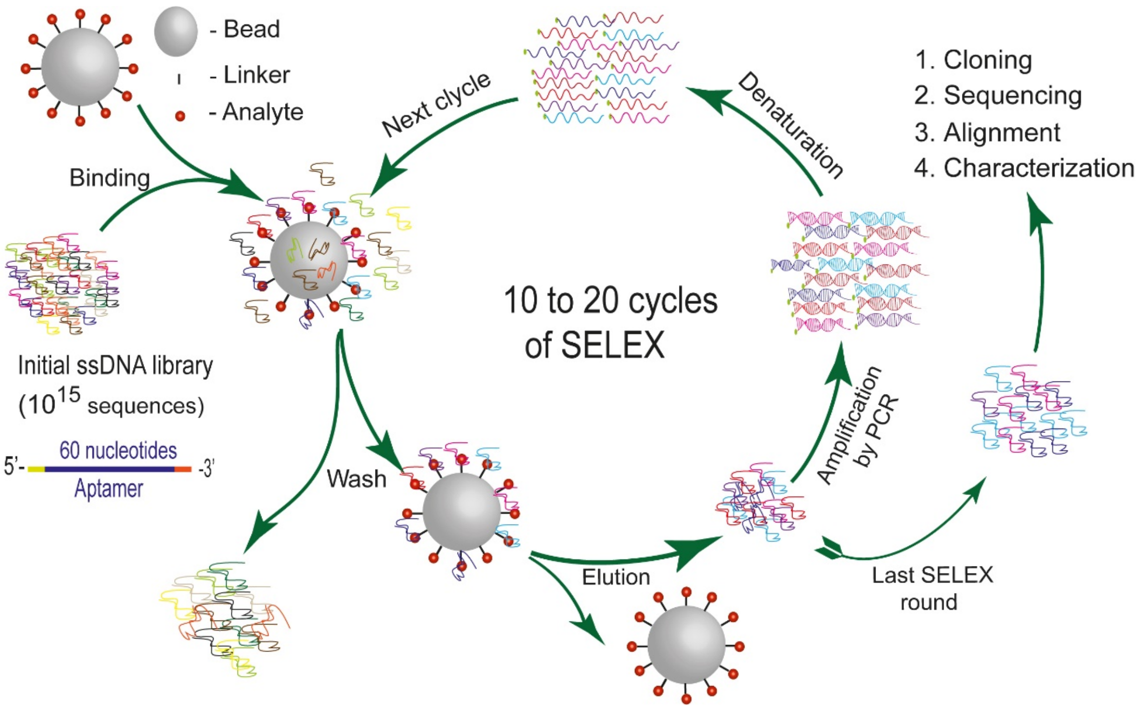

Supplementary Data Figure S6 Diagram of the SELEX protocol
